# Relationship of preseismic, coseismic, and postseismic fault ruptures of two large interplate aftershocks of the 2011 Tohoku earthquake with slow-earthquake activity

**DOI:** 10.1038/s41598-020-68692-x

**Published:** 2020-07-21

**Authors:** Hisahiko Kubo, Tomoaki Nishikawa

**Affiliations:** 1 National Research Institute for Earth Science and Disaster Resilience, 3-1, Tennodai, Tsukuba, Ibaraki 305-0006 Japan; 2 Disaster Prevention Research Institute, Kyoto University, Gokasho, Uji, Kyoto 611-0011 Japan

**Keywords:** Seismology, Tectonics

## Abstract

To improve our understanding of the interactions between regular and slow earthquakes along the Japan Trench, we investigated the spatial relationship of slow-earthquake activity with the preseismic, coseismic, and postseismic fault ruptures of interplate earthquakes off the Iwate and Ibaraki coasts, Japan, including two large interplate aftershocks of the 2011 Tohoku earthquake: the 2011 off Iwate earthquake (*M*_JMA_ 7.4) and the 2011 off Ibaraki earthquake (*M*_JMA_ 7.6). We found that the coseismic ruptures of these earthquakes did not overlap with the active areas of slow earthquakes, while their foreshocks and aftershocks occurred in slow-earthquake-prone areas. Moreover, the 2011 off Iwate earthquake and the previous M7-class events shared common fault rupture characteristics: coseismic rupture occurred in a common asperity area, and afterslip with many aftershocks was triggered in the active area of slow earthquakes. Off the Ibaraki coast, tremor activity on a subducting seamount located updip of the coseismic rupture of the 2011 off Ibaraki earthquake implies that the seamount acted as a soft barrier to the coseismic rupture of the 2011 off Ibaraki earthquake. This study demonstrates that large earthquakes off the Iwate and Ibaraki coasts feature similar rupture behaviors, spatially complementary distributions of coseismic ruptures with slow-earthquake activity and foreshock and aftershock activities within and around slow-earthquake-prone areas. This information is useful in considering future large earthquakes along the Japan Trench.

## Introduction

Over the past few decades, a considerable number of studies have been carried out on “slow earthquakes”, such as tectonic tremors, very-low-frequency earthquakes (VLFs), and slow slip events (SSEs)^1–5^. These studies have shown that slow earthquakes frequently occur in regions neighboring megathrust seismogenic zone and sometimes trigger large interplate earthquakes. Therefore, the investigation of slow-earthquake activity can provide insight into the earthquake-rupture mechanism in subduction zones. To improve our understanding of the interactions between regular and slow earthquakes in subduction zones, it is important to investigate the relationship between the fault ruptures of large interplate earthquakes and the slow-earthquake activity. Nishikawa et al.^5^ revealed the comprehensive spatial distribution of slow earthquakes along the Japan Trench and compared the resulting distribution with the coseismic rupture extents of the 2011 Tohoku earthquake and large interplate earthquakes that occurred before the 2011 Tohoku earthquake. The authors concluded that slow-earthquake activity spatially complements the coseismic ruptures of large interplate earthquakes and discussed the variation in fault slip behavior along the Japan Trench based on the regional characteristics of slow-earthquake activity.

To expand our understanding of the variation in fault slip behavior along the Japan Trench, we probe the relationship between the preseismic, coseismic, and postseismic fault ruptures of interplate earthquakes and the slow-earthquake activity off the Iwate and Ibaraki coasts, Japan. Although Nishikawa et al.^5^ focused mainly on coseismic fault ruptures, this study compiles various types of information on foreshock activity, mainshock coseismic rupture, aftershock activity, and slow-earthquake activity to understand the whole context of interplate slip behavior. We focus on two large interplate earthquakes that occurred after the 2011 Tohoku earthquake, namely, the 2011 off Iwate earthquake (*M*_JMA_ 7.4) and the 2011 off Ibaraki earthquake (*M*_JMA_ 7.6), which were not featured in Nishikawa et al.^5^. We investigate the spatial relationship of the slow-earthquake activity off the Iwate and Ibaraki coasts with the coseismic rupture extents of *M7*-class interplate earthquakes including the two large earthquakes in 2011. We further examine the spatial relationship of slow-earthquake activity with the foreshock and aftershock activities. The slow-earthquake observations employed herein include tremors, VLFs, and SSEs as well as earthquake swarms containing repeating earthquakes (referred to hereafter as “repeaters”), which can be used as potential indicators of SSEs^5–7^. We also discuss the role of a subducting seamount off the Ibaraki coast on the coseismic fault rupture of the 2011 off Ibaraki earthquake. Finally, we discuss the similarity between the rupture behaviors of large earthquakes off the Iwate and Ibaraki coasts.

### The 2011 off Iwate earthquake

**Figure 1:**
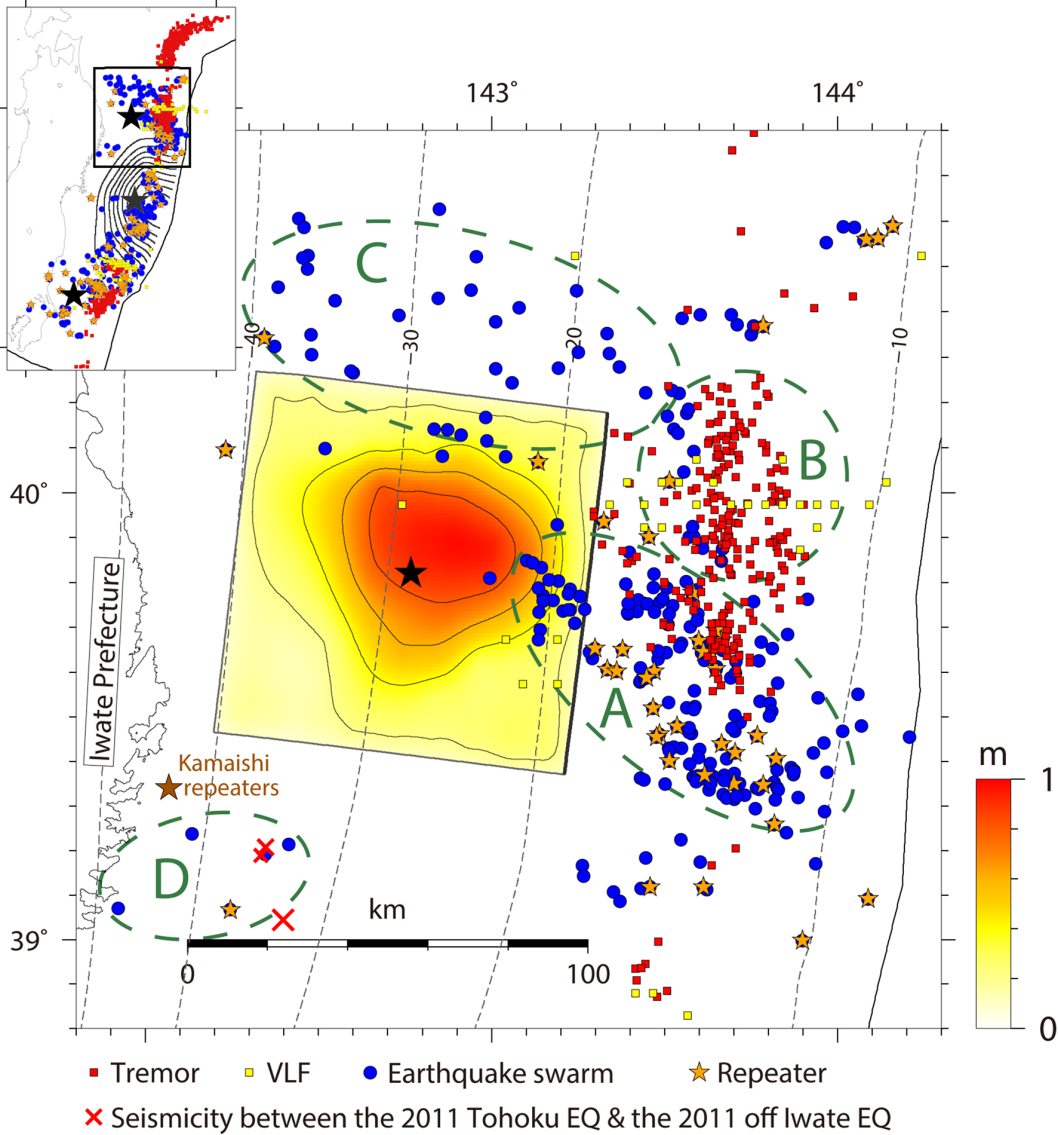
Comparison of the total slip distribution of the 2011 off Iwate earthquake estimated in this study (0.2 m contour interval) with the spatial distributions of slow earthquakes and other related observations^5^: tremors (red squares), VLFs (yellow squares), earthquake swarms (blue circles), and repeaters (orange stars). The black star indicates the epicenter of the 2011 off Iwate earthquake. The characteristic distributions of slow earthquakes are outlined by dark-green dashed circles. The red crosses denote the epicenters of the seismicity in the period between the 2011 Tohoku earthquake and the 2011 off Iwate earthquake. The brown star indicates the horizontal location of the repeating-earthquake sequence off Kamaishi^9,10^. The broken gray lines indicate the isodepth contours of the upper boundary of the Pacific plate 30 with a 10 km interval. The gray contours and gray star in the upper left inset indicate the total slip distribution of the 2011 Tohoku earthquake^41^ and its epicenter, respectively (contour interval of 4.3 m). This figure was rendered by Generic Mapping Tools (GMT)^42^ 4.5.14.

Off the coast of Iwate Prefecture, Tohoku region, Northeast Japan (Fig. 1), a thrust-type interplate earthquake with the Japan Meteorological Agency (JMA) magnitude (*M*_JMA_) of 7.4 occurred at 15:08 on March 11, 2011 [JST], approximately 20 min after the 2011 Tohoku earthquake. The moment magnitude (*M*_w_) estimated from the moment tensor inversion by F-net of National Research Institute for Earth Science and Disaster Resilience (NIED) was 7.4. This event was the second-largest interplate aftershock of the 2011 Tohoku earthquake. Nishikawa et al.^5^ divided the slow-earthquake activity along the Japan Trench into three along-strike segments: the northern segment (39° N to 40.7°N), the central segment (37.3°N to 39°N), and the southern segment (south of 37.3°N). This earthquake occurred in the northern segment.

In this study, we estimated the source process of this earthquake by using a joint source inversion with near-source waveforms and geodetic data (see “Methods” section). The source-inversion analysis revealed large slips (> 0.5 m) encompassing the epicenter at depths of 20–35 km with a maximum slip of 1.0 m, and their spatial extent was approximately 50 km × 60 km (Fig. 1).

Slow earthquakes actively occurred in the area surrounding the source region of the 2011 off Iwate earthquake (see “Methods” section). Tremors, VLFs, repeaters, and earthquake swarms were detected on the updip side of this event (areas A and B in Fig. 1), and earthquake swarms were also found north of this event (area C in Fig. 1). The area of large slip for the 2011 off Iwate earthquake did not reach the active area of slow earthquakes, indicating that the coseismic rupture of this event spatially complements the surrounding slow-earthquake activities.

**Figure 2:**
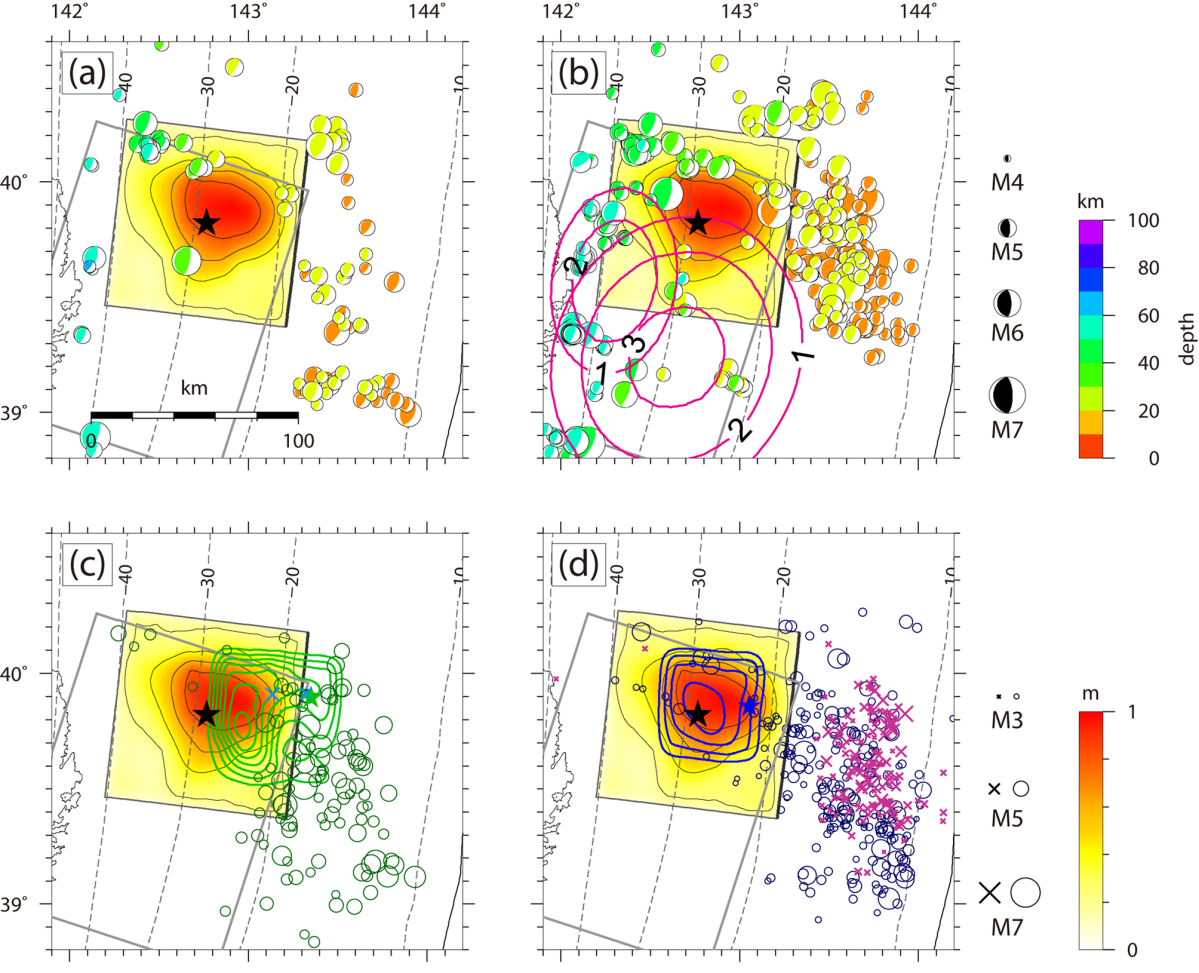
Map view of the total slip distribution of the 2011 off Iwate earthquake with the spatial distributions of focal mechanisms of interplate earthquakes in the period of (**a**) 1997—14:46 on 11 March 2011 [JST] (before the 2011 Tohoku earthquake) and (**b**) 15:15 on 11 March 2011 [JST]—2015 (after the 2011 off Iwate earthquake). These interplate earthquakes were selected from the F-net moment tensor catalog based on the following conditions: 155° ≤ strike ≤ 245°, 0° ≤ dip ≤ 40°, 45° ≤ rake ≤ 135°, 10 km ≤ depth ≤ 80 km, and *M*_w_ ≥ 4. The size of each focal mechanism is proportional to its moment magnitude. The magenta contours in (**b**) represent the distribution of cumulative afterslip values with a 1 m interval over 1 m for 5 years after the 2011 Tohoku earthquake^11^. The gray rectangle represents the Sanriku-oki low-seismicity region (SLSR)^16^. (**c**) Comparison with the total slip distribution of the 1960 far off Sanriku earthquake^12^ (green contour with a 0.2 m interval) and the spatial distributions of the foreshocks (4 h before the mainshock; cyan crosses) and aftershocks (1 month after the mainshock; dark green circles). (**d**) Comparison with the total slip distribution of the 1989 off Sanriku earthquake^12^ (blue contour with a 0.2 m interval) and the spatial distributions of foreshocks (5 days before the mainshock; purple crosses) and aftershocks (1 month after the mainshock; dark blue circles). This figure was rendered by GMT^42^ 4.5.14.

The spatial distributions of interplate earthquakes before the 2011 Tohoku earthquake and after the 2011 off Iwate earthquake are shown in Fig. 2a, b, respectively. In both periods, few interplate earthquakes occurred in the area of large slip for the 2011 off Iwate earthquake. The aftershocks mainly occurred on the updip side of this earthquake corresponding to the active area of earthquake swarms containing repeaters (area A in Fig. 1). According to the JMA unified seismic catalog^8^, three earthquakes occurred in the period between the 2011 Tohoku earthquake and the 2011 off Iwate earthquake (red crosses in Fig. 1) within and around area D, where an earthquake swarm containing a repeater was found in 1996. This area is also close to a typical repeating-earthquake sequence off Kamaishi, Northeast Japan^9,10^.

An analysis of geodetic land and seafloor measurements^11^ suggested that moderate deep afterslip occurred off the Iwate coast after the 2011 Tohoku earthquake (magenta contours in Fig. 2b). This afterslip region is located south and southwest of the source area of the 2011 off Iwate earthquake but does not greatly overlap with the coseismic slip area of this event.

Several *M*7-class large interplate earthquakes, including the 1960 far off Sanriku earthquake (*M*_JMA_ 7.2) and the 1989 off Sanriku earthquake (*M*_JMA_ 7.1 and *M*_w_ 7.4, Global CMT), occurred off the Iwate coast. The source inversion analysis^12^ using near-source waveforms indicated that the major slip area of the 1989 event was the same as that of the 1960 event; their slip distributions are shown in Fig. 2c,d, together with the slip distribution of the 2011 event. The moment magnitudes of the source models for the 1960 and 1989 events are 7.3 and 7.0, respectively. These figures indicate that the area of large slip for the 2011 event roughly overlaps with the area of large slip shared by the two previous events, suggesting that the common asperity area was ruptured in all three past earthquakes, although the epicenter location differed among these events.

The spatial distributions of the aftershocks corresponding to the 1960 and 1989 events are shown in Fig. 2c,d, respectively. The aftershock distributions are similar between the two events, with the active aftershock area located updip of their coseismic rupture areas^12^. Furthermore, the common aftershock area corresponds to the active aftershock area of the 2011 event, indicating that the three events have common fault rupture characteristics: coseismic rupture occurred in the common asperity and many aftershocks were triggered on the updip side of the asperity. However, the time interval between the 1989 and 2011 events is approximately two-thirds of that between the 1960 and 1989 events. The shortening of the recurrence interval may have been caused by the triggering effect of the 2011 Tohoku earthquake.

The common aftershock area corresponds to the active area of earthquake swarms containing repeaters (area A in Fig. 1). The occurrence rate of repeaters in this area increased following the 1989 event^13,14^ and the 2011 Tohoku earthquake^14^. Considering that the accelerated recurrence of repeaters reflects rapid aseismic slip on the plate interface^5–7^, these results suggest that afterslip occurred just after the 1989 and 2011 events and that these afterslip triggered numerous aftershocks on the updip side of the coseismic ruptures. The similarity between the aftershock activity of the 1960 event and that of the other two events implies that an aseismic slip transient also occurred immediately after the 1960 event in the same area.

In area A, many earthquake swarms containing repeaters sporadically occurred outside the postseismic periods of the three large interplate events. Bursts of repeaters and earthquake swarms were detected in 1991, 1992, 1996, 2008, 2015, and 2016 during the periods of 1999–2010 and 2014–2016^5^. Moreover, Kawasaki et al.^15^ suggested the occurrence of an “ultraslow earthquake” in this area in 1992; the event was a *M*_w_ 7.3–7.7 geodetically detected SSE accompanied by a swarm of seven *M*_JMA_ $\ge$ 6 interplate earthquakes. The above suggests that aseismic slip transients accompanied by repeaters and earthquake swarms frequently occurred in area A and were sometimes triggered by adjacent large interplate earthquakes.

The 1989 event was preceded by significant foreshock activity in area A (Fig. 2d), while only a few foreshocks occurred before the 1960 event (Fig. 2c). The difference in these foreshock activities might be related to the location of the hypocenters of these events. The epicenter of the 1960 event (with only a few foreshocks) was located at the edge of the active foreshock area of the 1989 event, while the epicenter of the 1989 event (with numerous foreshocks) was located closer to the asperity. In the case of the 2011 event, only a few foreshocks occurred in area D (Fig. 1); however, we cannot rule out the possibility that the disturbances of the 2011 Tohoku earthquake and its aftershocks caused other foreshocks to be overlooked.

Ye et al.^16^ proposed the existence of the Sanriku-Oki low-seismicity region (SLSR, gray rectangle in Fig. 2) on the northern margin of the 2011 Tohoku earthquake rupture zone; the SLSR lacks large historical earthquakes and features a relatively low level of moderate-magnitude earthquakes. However, the coseismic ruptures of the 1960, 1989, and 2011 events occurred in the northern part of the SLSR. Thus, *M*7-class interplate earthquakes recurrently ruptured the northern part of the SLSR, and hence, this region cannot be characterized by low seismicity. On the other hand, the central and southern parts of the SLSR may mainly slip aseismically, as suggested by the large afterslip of the 2011 Tohoku earthquake (Fig. 2b) and the high creep rate inferred from the repeaters off Kamaishi^9,10^.

### The 2011 off Ibaraki earthquake

**Figure 3:**
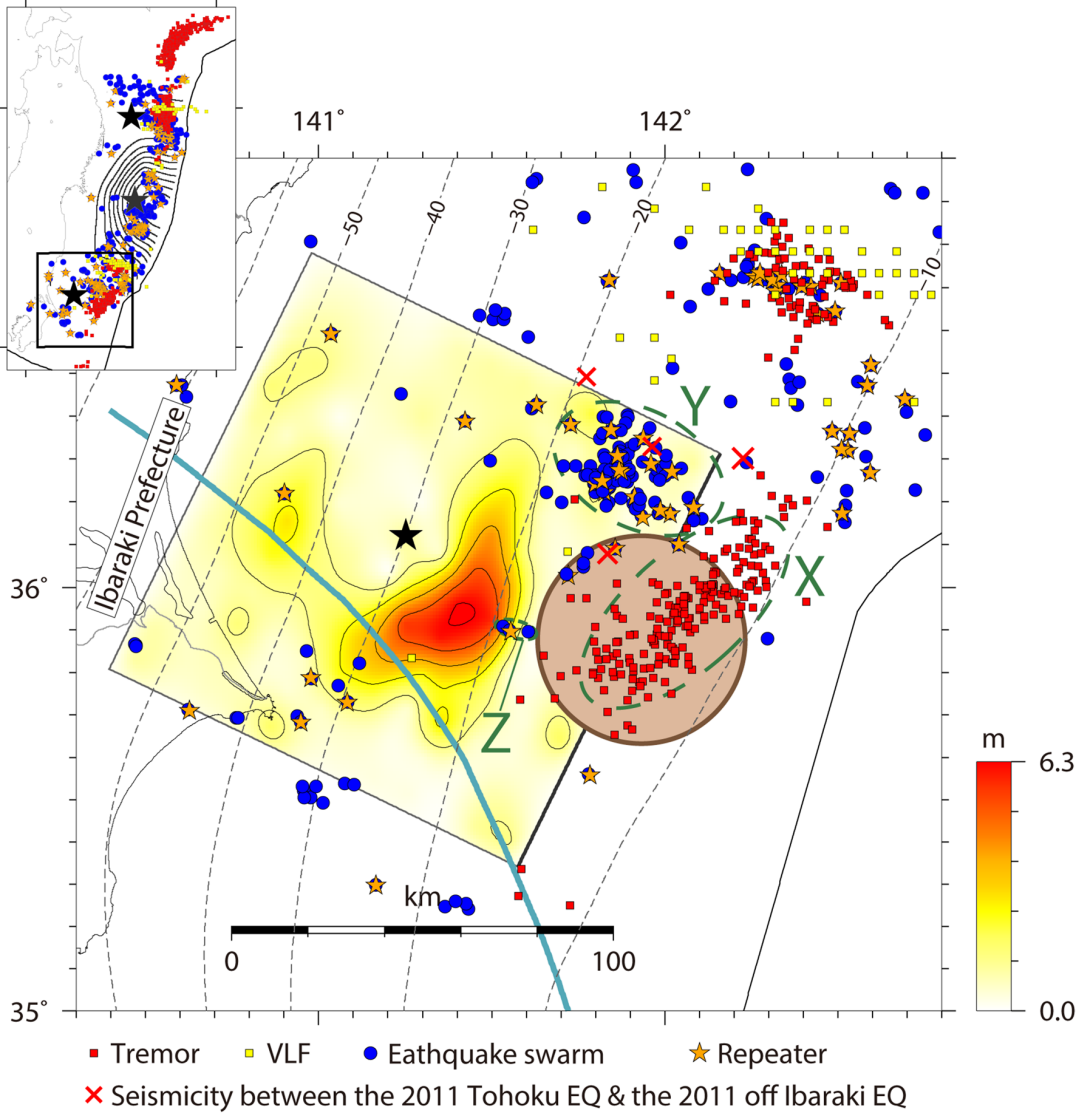
Comparison of the total slip distribution the 2011 off Ibaraki earthquake^17^ (1.26 m contour interval) with the spatial distributions of slow earthquakes and other related observations^5^: tremors (red squares), VLFs (yellow squares), earthquake swarms (blue circles), and repeaters (orange stars). The black star indicates the epicenter of the 2011 off Ibaraki earthquake. The characteristic distributions of slow earthquakes are outlined by dark-green dashed circles. The red crosses denote the epicenters of the seismicity in the period between the 2011 Tohoku earthquake and the 2011 off Ibaraki earthquake. The brown shaded zone indicates the subducting seamount^20^. The dark cyan line indicates the northeastern edge of the Philippine Sea plate^43^. This figure was rendered by GMT^42^ 4.5.14.

Off the coast of Ibaraki Prefecture, Kanto region, East Japan (Fig. 3), a thrust-type interplate earthquake of *M*_JMA_ 7.7 occurred at 15:15 on March 11, 2011 [JST], approximately 30 min after the 2011 Tohoku earthquake. The moment magnitudes estimated from the moment tensor inversion by GCMT and NIED F-net were 7.9 and 7.8, respectively. This event was the largest aftershock of the 2011 Tohoku earthquake that occurred in the southern segment of the Japan Trench based on the segmentation of Nishikawa et al.^5^.

Kubo et al.^17^ estimated the source process of this earthquake from near-source waveform records and geodetic data. The authors revealed that the area of large slip was located approximately 20 km southeast of the hypocenter (at depths of 20–30 km) and its spatial extent was approximately 60 km × 30 km. Figure 3 shows the total slip distribution of this event together with the slow-earthquake acitivity^5^. Tremors actively occurred updip of the area of large slip for the 2011 off Ibaraki earthquake (area X in Fig. 3), while earthquake swarms with repeaters actively occurred northeast of the area of large slip for the 2011 off Ibaraki earthquake (area Y in Fig. 3). These findings indicate that the coseismic rupture of this event does not overlap with the active area of slow earthquakes.

The seismicity surrounding the 2011 off Ibaraki earthquake has been investigated in previous studies^17–19^. The seismicity before and after the 2011 off Ibaraki earthquake was low in the area of large slip for this earthquake. Aftershocks actively occurred within and around area Y, which is characterized by many earthquake swarms containing repeaters (Fig. 3). Nakatani et al.^19^ further discovered additional aftershock activity in area Z in Fig. 3 through ocean-bottom seismic array observation. The authors also revealed that several earthquakes occurred in area Y and the region to the north of area Y in the period between the 2011 Tohoku earthquake and the 2011 off Ibaraki earthquake, as was partly recorded in the JMA unified seismic catalog (red crosses in Fig. 3), and that the highly active seismicity in area Y started after the occurrence of the 2011 off Ibaraki earthquake.

**Figure 4:**
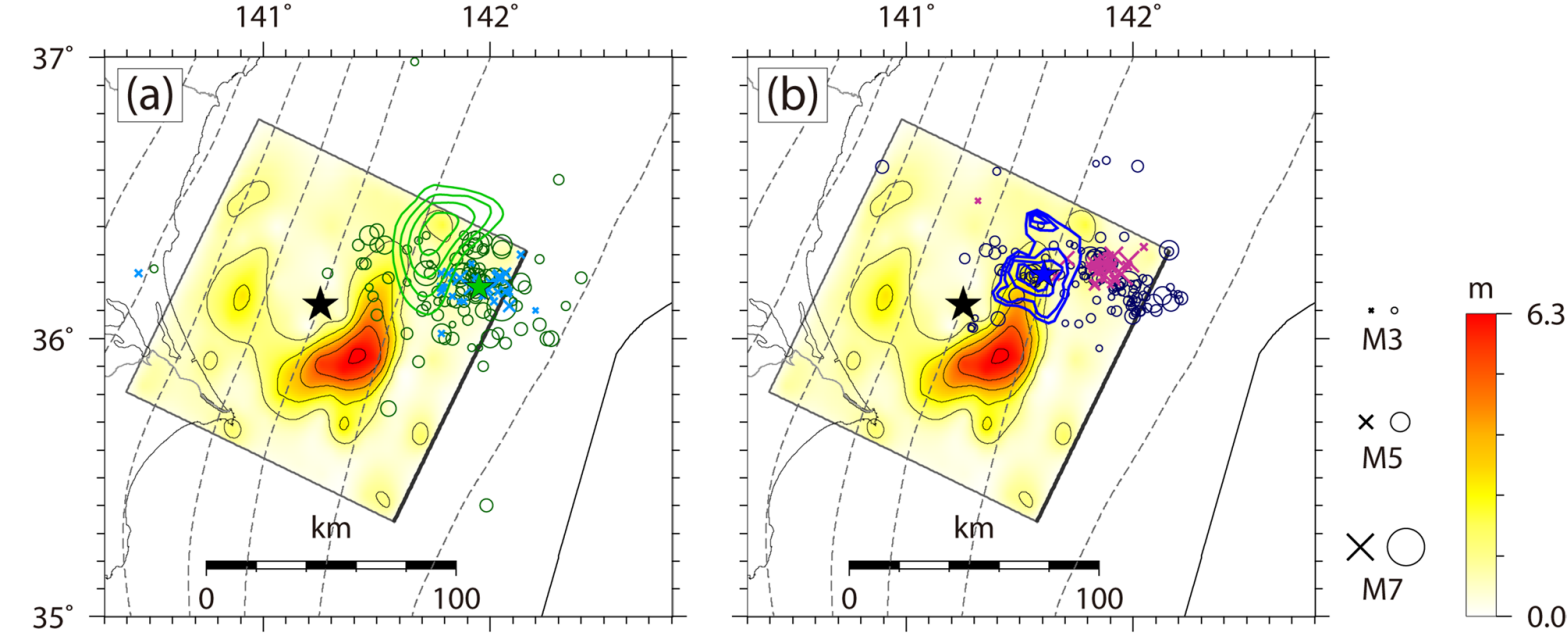
Map view of the total slip distribution of the 2011 off Ibaraki earthquake in comparison with (**a**) the total slip distribution of the 1982 off Ibaraki earthquake^20^ (green contour with a 0.1 m interval and a 0.4 m outermost contour), the epicenter of the 1982 off Ibaraki earthquake (green star), and the spatial distributions of foreshocks (3 days before the mainshock; cyan crosses) and aftershocks (1 month after the mainshock; dark green circles) and (**b**) the total slip distribution of the 2008 off Ibaraki earthquake^21^ (blue contour with a 0.3 m interval and a 0.6 m outermost contour), the epicenter of the 2008 off Ibaraki earthquake (blue star), and the spatial distributions of foreshocks (4 days before the mainshock; purple crosses) and aftershocks (1 month after the mainshock; dark blue circles). This figure was rendered by GMT^42^ 4.5.14.

The characteristic *M*7-class interplate earthquakes off the Ibaraki coast, including the 1982 off Ibaraki earthquake (*M*_JMA_ 7.0 and *M*_w_ 7.0, Global CMT) and the 2008 off Ibaraki earthquake (*M*_JMA_ 7.0, *M*_w_ 6.8, NIED F-net, and *M*_w_ 6.8, Global CMT), exhibit a recurrence interval of approximately 20 years^7,17,20^. The slip distributions of the two most recent events in 1982^20^ and 2008^21^ are shown in Fig. 4a, b, respectively, together with the slip distribution of the 2011 event. The moment magnitudes of the source models for the 1960 and 1989 events are 7.1 and 6.9, respectively. Their rupture areas roughly overlap and are located northeast of the area of large slip for the 2011 off Ibaraki earthquake. In addition, there was no slow-earthquake activity in the main coseismic rupture areas of the 1982 and 2008 events. Their intensive foreshock activities occurred in the active area of earthquake swarms containing repeaters (area Y), which is located updip of the main coseismic rupture area. Based on the history of repeaters and earthquake swarms, Nishikawa et al.^7^ suggested the recurrence of aseismic slip transients in this area and pointed out that the aseismic slip transients that preceded the 1982 and 2008 *M*7-class events had larger seismic moments than the other transients. Moreover, the aftershocks of the 1982 and 2008 events occurred mainly in area Y and the foreshocks and aftershocks of the 1982, 2008, and 2011 events occurred in this common area (area Y), but the coseismic rupture extent of the 2011 event does not overlap with those of the other events.

In the updip side of the source area of the 2011 off Ibaraki earthquake, a subducting seamount (brown circle in Fig. 3) was discovered by an active source seismic survey using ocean-bottom seismometers^20^. Based on the fact that the fault rupture of this earthquake did not reach the seamount zone, Kubo et al.^17^ suggested that the seamount likely stopped the rupture propagation of this event by acting as a barrier to fault rupture. However, the behavior of a seamount or ridge in resisting the coseismic rupture of an interplate earthquake is still controversial^17,20,22–25^. Here, we shall focus on the barrier mechanism of a seamount in response to fault rupture. One reasonable hypothesis is a “hard” barrier to fault rupture that has a strong frictional resistance. Scholz and Small^22^ suggested that the subduction of a large seamount increases the normal stress across the subduction interface and locally strengthens the seismic coupling (the frictional resistance). Accordingly, if the seamount area is not ready for coseismic rupture, a seamount with a strong frictional resistance will act as an indenter against the subducting plate and impedes earthquake rupture propagation. In Central America, the Cocos Ridge is expected to act as an indenter against the Caribbean Plate^23,24^. However, the hard-barrier interpretation in our case is inconsistent with the fact that the seamount zone overlaps the active area of tremors (area X) because the occurrence of tremors indicates a frequent release of interplate strain. Another plausible idea is a “soft” barrier to fault rupture that has little potential for coseismic fault ruptures. Mochizuki et al.^20^ suggested that the interplate coupling in the seamount zone is very weak, probably due to the fluid-rich sediment entrained in the subducting seamount. This indicates that the seamount has little potential for coseismic fault rupture and thus arrests coseismic rupture propagation. The occurrence of tremors supports the soft-barrier idea because abundant fluids are one of the possible causes of slow earthquakes^5,26^. Based on these observations, we propose that the fault rupture of the 2011 off Ibaraki earthquake was stopped by a soft-barrier seamount. Another example of the soft-barrier model is the Nazca Ridge, which is subducting beneath offshore central and southern Peru. Perfettini et al.^25^ revealed that the subducting Nazca Ridge with low interseismic coupling stopped the seismic rupture propagation of the 2007 Pisco earthquake (*M*_w_ 8.0) and featured prominent afterslip after the 2007 event.

### Similarity between the rupture behaviors of large earthquakes off the Iwate and Ibaraki coasts

The Iwate and Ibaraki offshore regions are seismically similar in two respects. First, the slow-earthquake activities are distributed updip of the coseismic slip areas of large interplate earthquakes without spatially overlapping. The shallow parts (shallower than 20 km) of the megathrust seismogenic zones off the Iwate and Ibaraki coasts frequently release their strain energy via slow earthquakes and do not rupture coseismically (Figs. 1, 3), consistent with the idea that slow-earthquake-prone areas hinder the coseismic rupture propagation of large interplate earthquakes^5^. Furthermore, abundant fluids entrained along an undulating plate interface, such as a subducting seamount, may facilitate slow deformation rather than fast coseismic rupture.

Second, the foreshock and aftershock activities of large interplate earthquakes occurred mostly within and around the slow-earthquake-prone areas off the Iwate and Ibaraki coasts, implying that these foreshocks and aftershocks are related to aseismic slip transients. Previous studies attributed the occurrence of intensive foreshock activity to SSEs preceding large interplate earthquakes^6,7^. Here, we speculate that some large interplate earthquakes off the Iwate and Ibaraki coasts (e.g., the 1989 off Sanriku and 1982 and 2008 off Ibaraki earthquakes) were preceded by SSEs located updip of their coseismic rupture areas. However, a large observable SSE that triggers intensive foreshock activity is not a prerequisite for the occurrence of large interplate earthquakes off the Iwate and Ibaraki coasts because the 1960 off Sanriku earthquake was preceded by only a few foreshocks (Fig. 2c).

This study clarified the relationship between slow earthquakes and the coseismic rupture extents of large interplate earthquakes off the Iwate and Ibaraki coasts along the Japan Trench. The spatially complementary distributions of slow earthquakes and the coseismic ruptures of large interplate earthquakes suggest that revealing the detailed distributions of slow earthquakes in subduction zones can improve our estimates of rupture extents for future great interplate earthquakes. Furthermore, the intensive foreshock activities of large interplate earthquakes within and around slow-earthquake-prone areas imply that monitoring the seismicity in the vicinity of slow-earthquake-prone areas is important for detecting possible precursors of impending interplate earthquakes.

## Methods

### Source inversion of the 2011 off Iwate earthquake

The source rupture process of the 2011 off Iwate earthquake was estimated by a joint source inversion of near-source strong-motion waveform data and static displacement data.

For the strong-motion data, we used three-components waveform records at 20 stations of NIED K-NET, KiK-net^27,28^ (Figures S1 and S2): 4K-NET stations on ground surface and 16 KiK-net stations in borehole. The original acceleration waveforms were numerically integrated in the time domain into velocity. The velocity waveforms were band-pass filtered between 0.02 and 0.2 Hz, resampled to 2 Hz, and windowed from 10 s before the S-wave arrival for 80 s.

The GPS data used in the source inversion were recorded by the GNSS Earth Observation Network (GEONET). We obtained station positions every 30 s using Kinematic Precise Point Positioning as implemented in RTKLIB^29^. To avoid the effects of the 2011 Tohoku earthquake, other aftershocks, and afterslip, the preseismic and postseismic positions were obtained by averaging the positions from 15:05:30 to 15:08:30 (JST) and from 15:11:00 to 15:14:00 (JST), respectively. Then, the coseismic static displacements for the event were obtained by differencing the preseismic and postseismic positions. This study used two horizontal displacement components (east–west and north–south) at 83 GEONET stations (Figure S3).

A curved fault model with a size of 90 km along strike and 90 km along dip (Figure S1) was assumed based on the shape of the plate boundary in the Japan Integrated Velocity Structure Model (JIVSM) Version 1^30^. The fault model was divided into 81 subfaults, each with dimensions of approximately 10 km × 10 km. The strike and dip angles of each subfault were calculated by Shiono’s^31^ method from grid data for the plate boundary of the JIVSM. The horizontal location of the rupture starting point was fixed at (142.7668° E, 39.8207° N), which is the epicenter location in the JMA unified seismic catalog. The depth of the rupture starting point was set to 28.6 km, which corresponds to the depth of the plate boundary in the JIVSM at the JMA epicenter.

The spatiotemporal rupture history was estimated by using the fully Bayesian multiple-time-window source inversion^32^. Because we estimated the weights of the slips with 45° and 135° rake angles for each time window at each subfault, together with a nonnegative constraint, the rake angle of the slip vector for each subfault was allowed to vary within 90° ± 45°. The slip history at each subfault was represented by a series of nine smoothed-ramp functions with a width of 4.0 s, each with a 2.0-s lag. The triggering velocity of the first time window of 3.6 km/s was selected to minimize the data-fit residual. Although the amplitudes of the strong-motion waveforms in the source inversion were normalized by the maximum amplitude at each station, this normalization was not applied to the geodetic data to avoid the instability caused by the low signal-to-noise ratio data with small absolute values^33^. The relative weight between the datasets was determined to ensure that the data fit for each dataset was satisfactory.

The strong-motion Green’s functions were calculated using the discrete wave number method^34^ and the reflection/transmission matrix method^35^, with a one-dimensional (1-D) layered velocity structure model. The 1-D velocity structure models were obtained for each station from the three-dimensional velocity structure model^36,37^. Logging data were also referenced for the KiK-net stations. For the geodetic Green’s functions, we calculated the theoretical static displacements caused by a unit slip on each subfault, assuming a homogeneous elastic half-space^38^.

From the posterior probability distributions of the slip at each subfault for each time window based on the 80,000 ensembles of a source model produced by the fully Bayesian multiple-time-window source inversion, we obtained the optimal source model composed of the median slips of the distributions. Figures S4, S5, and S6 show the total slip distribution of the 2011 off Iwate earthquake, the rupture progression, and the slip rate function at each subfault, respectively. The seismic moment and maximum slip of the estimated source model are 1.3 × 10^20^ N m (*M*_w_ 7.4) and 1.0 m, respectively. Large slips (> 0.5 m) are distributed at depths of 20–35 km including the epicenter with a maximum slip of 1.0 m, and the spatial extent of large slips is approximately 50 km × 60 km. During the first 5 s, the rupture slowly grew around the hypocenter with small slips. Subsequently, the rupture developed in the area of large slip with a large moment release between 5 and 20 s. The total rupture duration was approximately 30 s. The synthetic strong-motion waveforms and static displacements matched well the observations (Figures S2 and S3).

### Slow-earthquake catalogs

We used the catalogs of tectonic tremors, VLFs, and earthquake swarms containing repeaters provided by Nishikawa et al.^5^ The tectonic tremor catalog covers the period from August 2016 to August 2018, and the VLF catalog covers the period from January 2005 to August 2018. The catalog of earthquake swarms containing repeaters off the Iwate coast covers the periods from January 1991 to December 2010, and from January 2014 to August 2016, while catalog of earthquake swarms containing repeaters off the Ibaraki coast covers the period from January 1991 to December 2010. In the catalogs, earthquake swarms are classified into “background swarms”, where the first event is a background event, and “aftershock swarms”, where the first event is an aftershock of a preceding earthquake. Nishikawa et al.^5^ considered a background swarm to be a typical earthquake swarm, while they considered an aftershock swarm to be an anomalously intensive aftershock sequence that does not obey the Omori-Utsu law^39^. This classification was disregarded in this study because it occasionally results in an obvious misclassification. For example, a swarm of seven *M*_JMA_ ≥ 6 interplate earthquakes accompanying the 1992 off Sanriku ultraslow earthquake^15^, which was a typical earthquake swarm^13^, was falsely classified as an aftershock swarm in the catalog. The classification criterion, which depends solely on the first event of each earthquake swarm sequence, seems to occasionally fail when the magnitude of events in an earthquake swarm is relatively large (*M* ≥ 6).

## Supplementary information

Supplementary information.

## Data Availability

Ground motion records at NIED K-NET, KiK-net^28^ are available at https://www.kyoshin.bosai.go.jp/. GPS data recorded by GEONET of the Geospatial Information Authority of Japan are available at https://terras.gsi.go.jp/. The moment tensor solution catalog of NIED F-net^40^ is available at https://www.fnet.bosai.go.jp/. The focal mechanism catalog of the Global CMT is available at https://www.globalcmt.org/. The JMA unified seismic catalog^8^ is available at https://www.data.jma.go.jp/svd/eqev/data/bulletin/index_e.html. The subsurface structure model for Japan is available from NIED J-SHIS^37^ at https://www.j-shis.bosai.go.jp/. JIVSM^30^ is available at https://www.jishin.go.jp/evaluation/seismic_hazard_map/lpshm/12_choshuki_dat/. RTKLIB^29^ is available at https://www.rtklib.com/.
