# Supplementary material for: Relationship of preseismic, coseismic, and postseismic fault ruptures of two large interplate aftershocks of the 2011 Tohoku earthquake with slow-earthquake activity
Source: Sci Rep. 2020 Jul 21;10:12044. doi: 10.1038/s41598-020-68692-x (PMC7374571; doi:10.1038/s41598-020-68692-x)
Supplement: Supplementary file 1 — Supplementary information. [file 41598_2020_68692_MOESM1_ESM.docx]

**Additional files for**

**Spatial relationship between rupture area of two large inter-plate aftershocks of the 2011 Tohoku earthquake and slow-earthquake activity**

Author #1: Hisahiko Kubo, National Research Institute for Earth Science and Disaster Resilience, 3-1, Tennodai, Tsukuba, Ibaraki 305-0006, Japan, hkubo@bosai.go.jp

Author #2: Tomoaki Nishikawa, Disaster Prevention Research Institute, Kyoto University, Gokasho, Uji, Kyoto 611-0011, Japan, nishikawa.tomoaki.68s@st.kyoto-u.ac.jp

Corresponding author: Hisahiko Kubo, National Research Institute for Earth Science and Disaster Resilience, 3-1, Tennodai, Tsukuba, Ibaraki 305-0006, Japan, hkubo@bosai.go.jp

Content of this file: Figs. S1, S2, S3, S4, S5, and S6


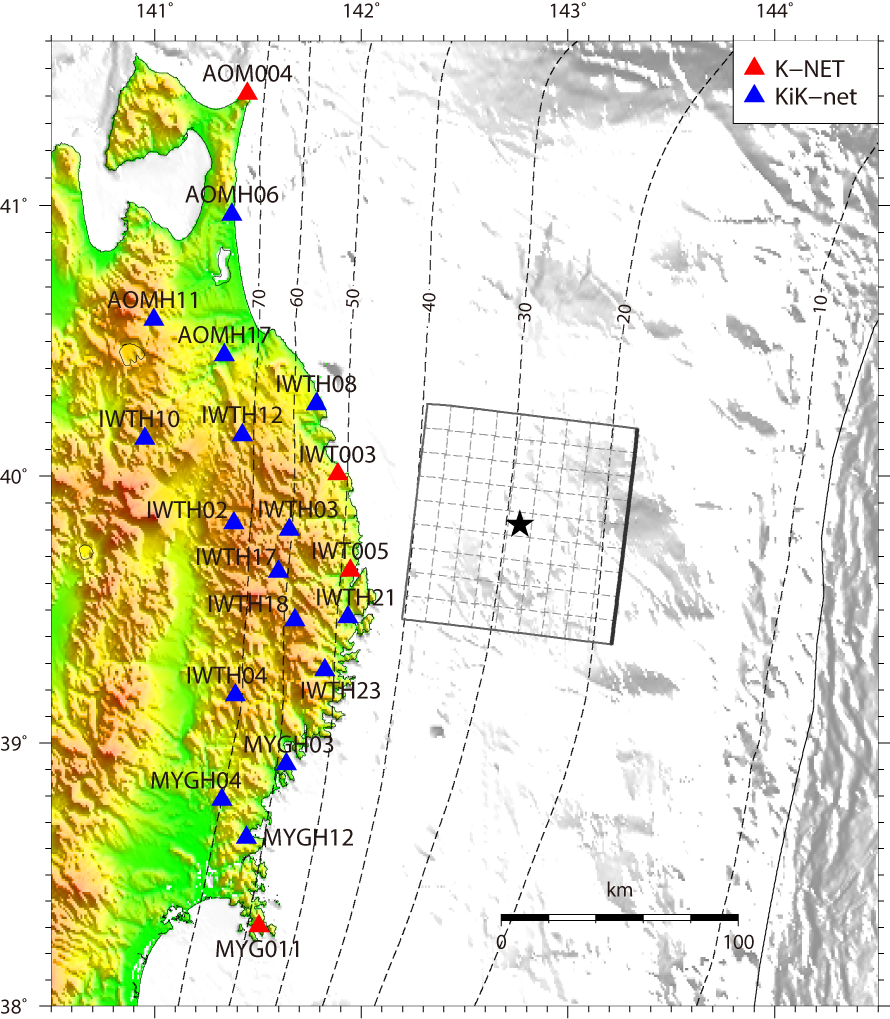


Fig. S1

Map of the study area for the 2011 off Iwate earthquake. The black star denotes the epicenter of the 2011 off Iwate earthquake. The black line denotes the outline of the fault model used in the source inversion. The triangles indicate the stations used in the source inversion. The color of the triangles indicates the observation network: blue for KiK-net, and red for K-NET. The broken gray lines indicate the isodepth contours of the plate boundary in the JIVSM^30^ with a 10 km interval. This figure was rendered by GMT^42^ 4.5.14.


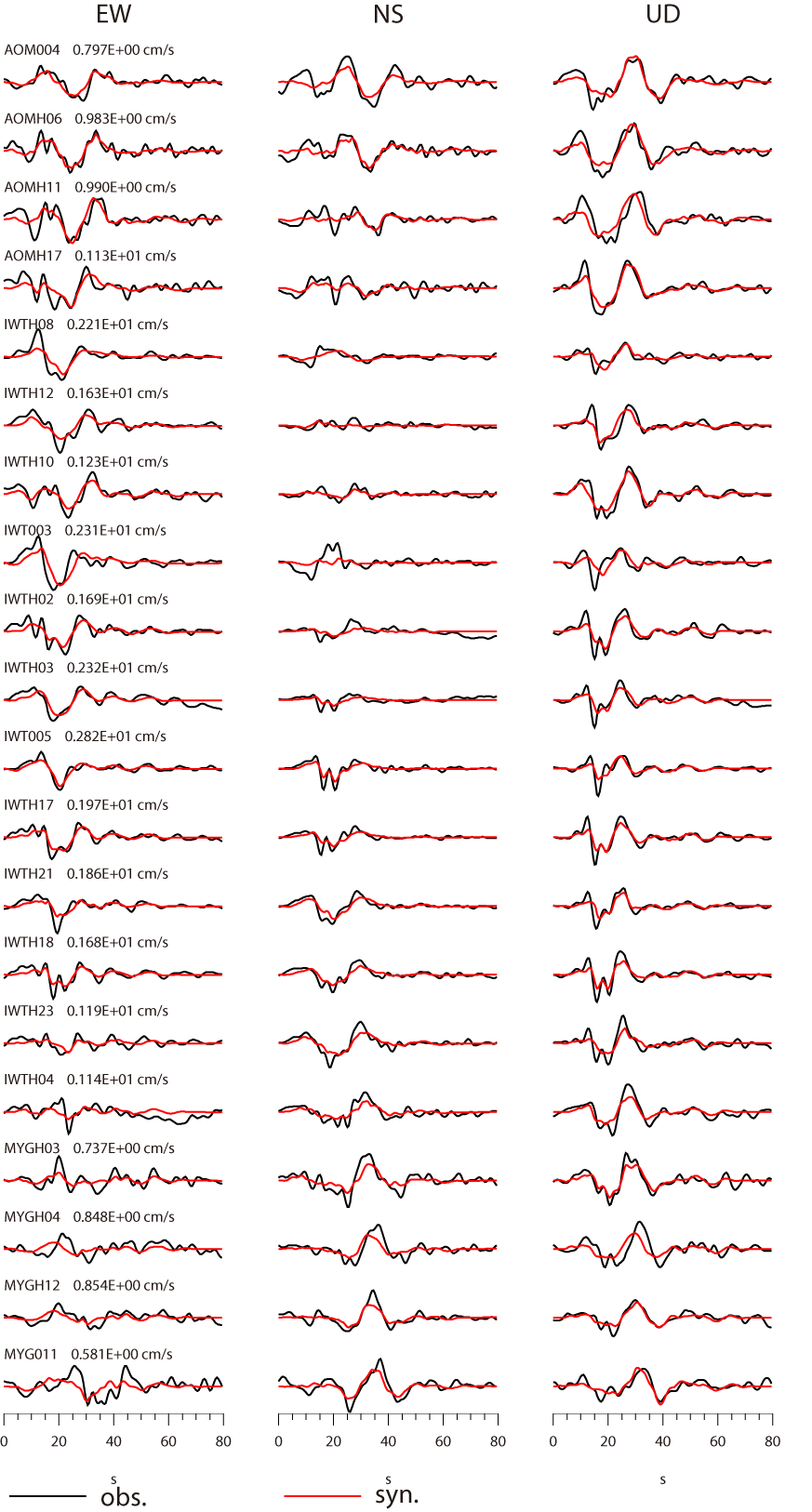


Fig. S2

Comparison between the observed waveforms (black) and synthetic waveforms (red) in the source inversion of the 2011 off Iwate earthquake. The maximum amplitudes of the observed waveforms are shown in the upper left corner of each waveform. This figure was rendered by GMT^42^ 4.5.14.


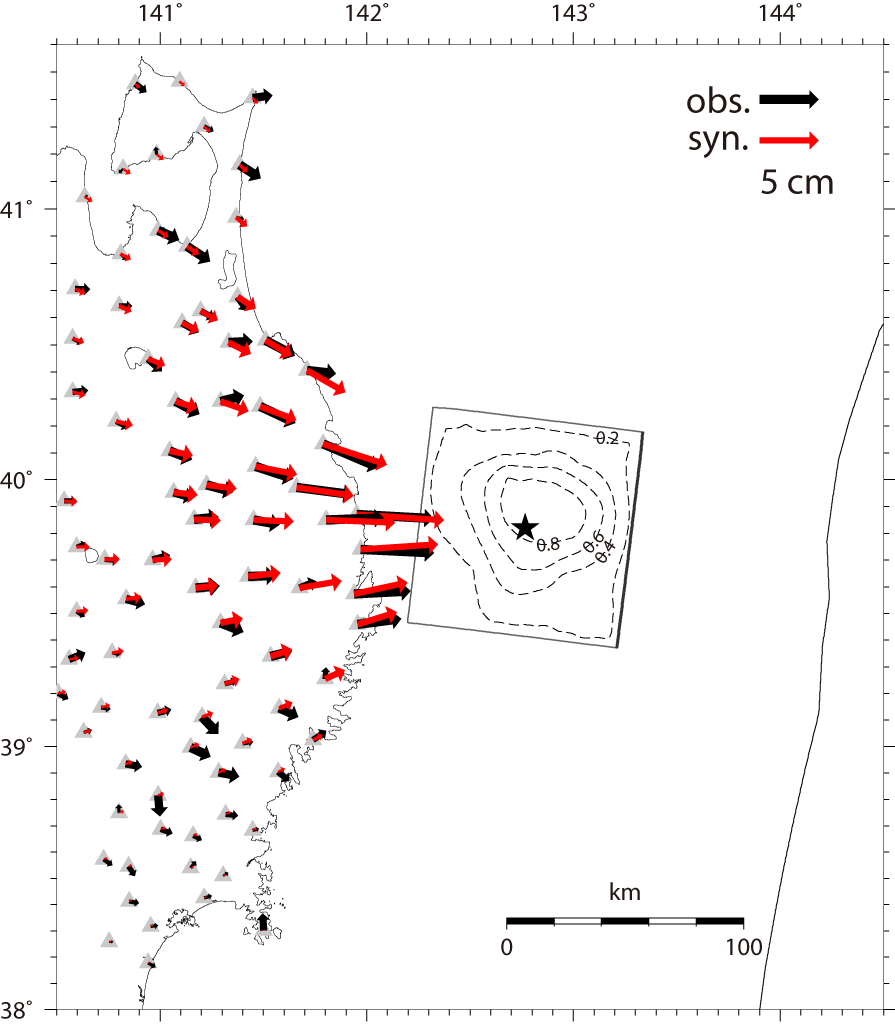


Fig. S3

Comparison between the observed (black) and synthetic (red) static horizontal displacements in the source inversion of the 2011 off Iwate earthquake. The broken contours indicate the estimated slip distribution. The black star denotes the epicenter. This figure was rendered by GMT^42^ 4.5.14.


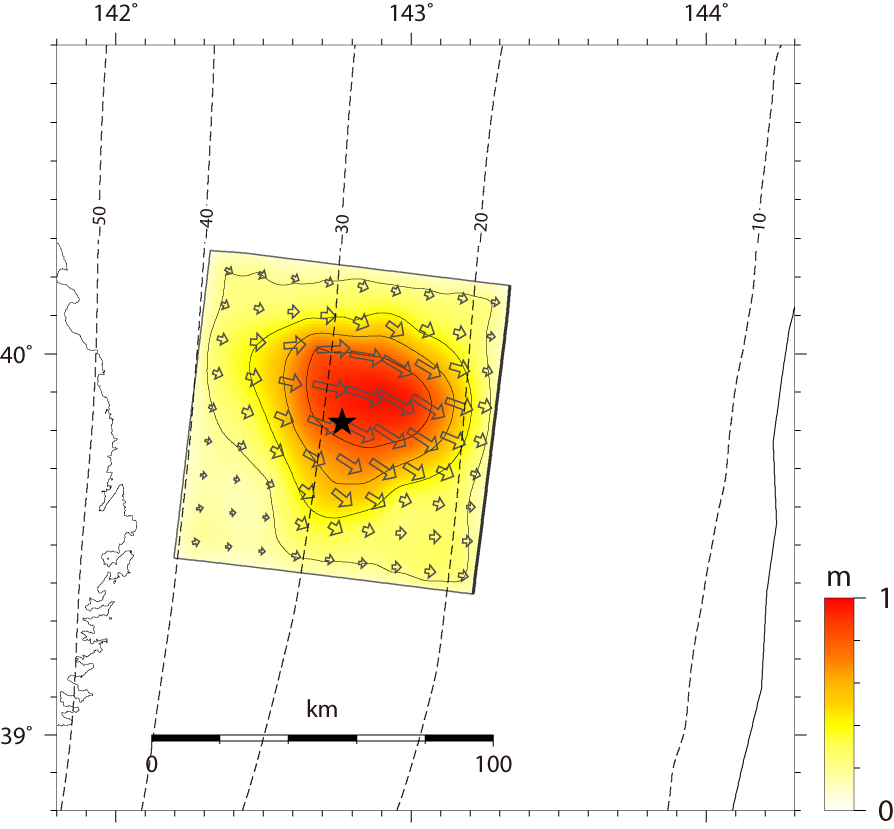


Fig. S4

Map projection of the total slip distribution of the 2011 off Iwate earthquake. The slip contour interval is 0.2 m. The star denotes the epicenter. Vectors denote the direction and the amount of slip on the hanging wall side. This figure was rendered by GMT^42^ 4.5.14.


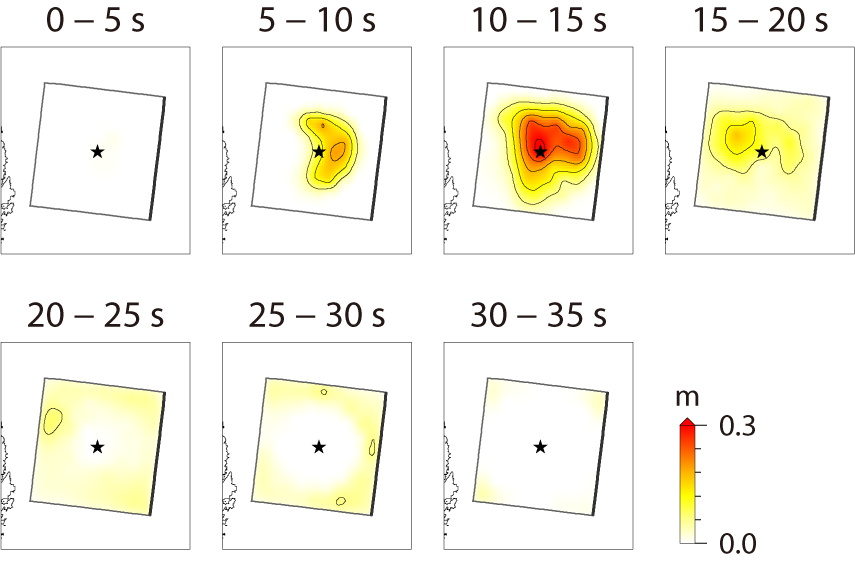


Fig. S5

Rupture progression of the 2011 off Iwate earthquake in terms of the amount of slip during each 5.0-s time window. The slip contour interval is 0.6 m. The star denotes the epicenter. This figure was rendered by GMT^42^ 4.5.14.


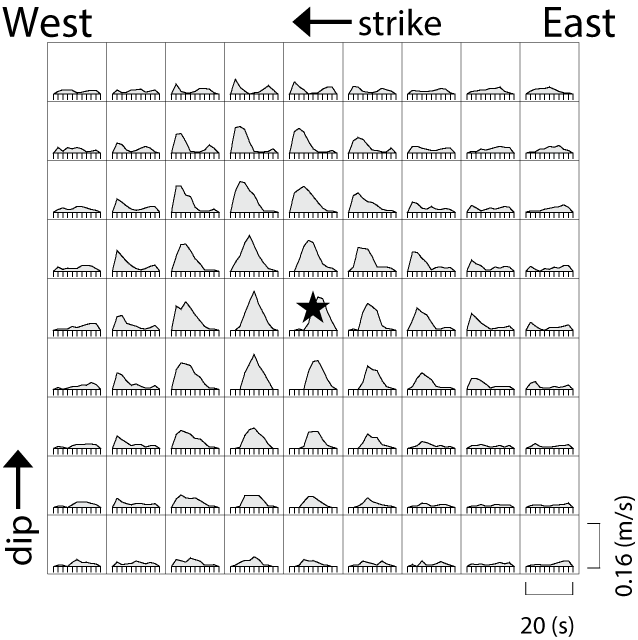


Fig. S6

Source time function for each subfault of the 2011 off Iwate earthquake. The star denotes the subfault corresponding to the subfault on which the rupture initiated. This figure was rendered by GMT^42^ 4.5.14.
